# Predominantly Pro-Inflammatory Phenotype with Mixed M1/M2 Polarization of Peripheral Blood Classical Monocytes and Monocyte-Derived Macrophages among Patients with Excessive Ethanol Intake

**DOI:** 10.3390/antiox12091708

**Published:** 2023-09-01

**Authors:** María Fernández-Regueras, Cristina Carbonell, Daniel Salete-Granado, Juan-Luis García, Marcos Gragera, María-Ángeles Pérez-Nieto, Francisco-Javier Morán-Plata, Andrea Mayado, Jorge-Luis Torres, Luis-Antonio Corchete, Ricardo Usategui-Martín, Elena Bueno-Martínez, Maura Rojas-Pirela, Guadalupe Sabio, Rogelio González-Sarmiento, Alberto Orfao, Francisco-Javier Laso, Julia Almeida, Miguel Marcos

**Affiliations:** 1 Hospital Universitario de Burgos, 09006 Burgos, Spain; 2 Hospital Universitario de Salamanca, 37007 Salamanca, Spain; 3 Instituto de Investigación Biomédica de Salamanca (IBSAL), 37007 Salamanca, Spain; 4 Departamento de Medicina, Universidad de Salamanca, 37007 Salamanca, Spain; 5 Translational and Clinical Research Program, Centro de Investigación del Cáncer e Instituto de Biología Molecular y Celular del Cáncer (IBMCC), 37007 Salamanca, Spain; 6 Centro Nacional de Biotecnología, Consejo Superior de Investigaciones Científicas, 28049 Madrid, Spain; 7 Centro Nacional de Investigaciones Cardiovasculares, 28029 Madrid, Spain; 8 Fundación Instituto de Estudios de Ciencias de la Salud de Castilla y León, 42002 Soria, Spain; 9 Biomedical Research Networking Centre Consortium of Oncology (CIBERONC), Instituto de Salud Carlos III, 28029 Madrid, Spain; 10 Complejo Asistencial de Zamora, 49022 Zamora, Spain; 11 Departamento de Biología Celular, Facultad de Medicina, Universidad de Valladolid, 47005 Valladolid, Spain

**Keywords:** alcohol use disorders, excessive alcohol drinkers, monocyte/macrophage phenotype, M1, M2, innate immunity, flow cytometry, microRNA

## Abstract

Excessive alcohol consumption impairs the immune system, induces oxidative stress, and triggers the activation of peripheral blood (PB) monocytes, thereby contributing to alcoholic liver disease (ALD). We analyzed the M1/M2 phenotypes of circulating classical monocytes and macrophage-derived monocytes (MDMs) in excessive alcohol drinkers (EADs). PB samples from 20 EADs and 22 healthy controls were collected for isolation of CD14+ monocytes and short-term culture with LPS/IFNγ, IL4/IL13, or without stimulation. These conditions were also used to polarize MDMs into M1, M2, or M0 phenotypes. Cytokine production was assessed in the blood and culture supernatants. M1/M2-related markers were analyzed using mRNA expression and surface marker detection. Additionally, the miRNA profile of CD14+ monocytes was analyzed. PB samples from EADs exhibited increased levels of pro-inflammatory cytokines. Following short-term culture, unstimulated blood samples from EADs showed higher levels of soluble TNF-α and IL-8, whereas monocytes expressed increased levels of surface TNF-α and elevated mRNA expression of pro-inflammatory cytokines and inducible nitric oxide synthase. MDMs from EADs showed higher levels of TNF-α and CD206 surface markers and increased IL-10 production. LPS/IFNγ induced higher mRNA expression of Nrf2 only in the controls. miRNA analysis revealed a distinctive miRNA profile that is potentially associated with liver carcinogenesis and ALD through inflammation and oxidative stress. This study confirms the predominantly pro-inflammatory profile of PB monocytes among EADs and suggests immune exhaustion features in MDMs.

## 1. Introduction

Ethanol intake impairs both innate and adaptive immunity, with specific alterations detected depending on several factors, such as the pattern of ethanol intake and the presence of alcoholic liver disease (ALD) [1]. Among the wide array of alterations in the immune system displayed by patients with chronic and heavy ethanol intake, several authors have reported a pro-inflammatory response with increased production of inflammatory cytokines [2]. In particular, several circulating cells, such as peripheral blood (PB) monocytes and dendritic cells, have shown altered secretion profiles or increased mRNA levels of inflammatory cytokines [3,4].

This is particularly important because the activation of peripheral monocytes and the accumulation of macrophages in the liver are relevant in the pathogenesis of alcoholic liver disease [5]. In addition, the secretion of inflammatory cytokines mediated by the lipopolysaccharide (LPS)-toll-like receptor 4 (TLR4) pathway, such as tumor necrosis factor (TNF)-α by Kupffer cells [6,7], plays a pivotal role in the pathogenesis of ALD.

The key role of the mononuclear phagocyte system in alcohol-related liver damage suggests that macrophage polarization may be relevant to tissue injury during chronic alcohol consumption through several mechanisms, including oxidative stress. Briefly, macrophages may polarize toward pro- or anti-inflammatory phenotypes in response to different stimuli. When stimulated by LPS or Th1-secreted cytokines, macrophages show the so-called “classical” or M1 profile, which is characterized by the secretion of pro-inflammatory cytokines and the generation of reactive species. In turn, Th2-secreted cytokines such as IL-10, IL-4, and IL-13 result in the “alternative”, or M2 profile, which is characterized by the production of anti-inflammatory cytokines and promotion of tissue repair and wound healing [8,9]. In addition, regulators of antioxidant responses, such as Nrf2, can inhibit the M1 polarization and promote the M2 phenotype [10,11], and several miRNAs have been shown to modulate monocyte/macrophage polarization with potential effects on inflammation and other pathways related with liver damage [12].

While M1/M2 polarization has been extensively studied in other pathological conditions, e.g., tumor microenvironment, obesity, or chronic (autoimmune) inflammatory disorders, little is known regarding M1/M2 polarization in patients with chronic ethanol intake. Preliminary data from murine models of ALD [13,14,15] and blood samples from excessive drinkers [16] suggest that ethanol effects on macrophage polarization are apparently contradictory, as it may increase the expression of both M1- and M2-related markers, as well as of both pro- and anti-inflammatory mediators. The aim of this study was to analyze the inflammatory response of classical (CD14+) monocytes in the peripheral blood of patients with excessive alcohol consumption and to study the in vitro induced polarization of monocyte-derived macrophages.

## 2. Materials and Methods

### 2.1. Patients and Healthy Subjects

We included 20 patients with alcohol use disorder according to the DSM-5 criteria [17], who were referred to the Alcoholism Unit of the University Hospital of Salamanca. All patients included in this group had a pattern of chronic daily heavy ethanol intake and actively drank (≥90 g ethanol/day) for at least 5 years before they entered the study. All patients had normal prothrombin time, hemoglobin concentration, and serum albumin levels, and they were negative for hepatitis B surface antigen and antibodies to hepatitis C virus. They did not have other chronic or acute conditions that could alter the results of the study or were polydrug abusers. Patients with advanced liver disease were excluded based on clinical, analytical, and ultrasonographic studies, that is, individuals displaying physical stigmata of chronic liver disease (e.g., cutaneous signs, hepatosplenomegaly, gynecomastia, testicular atrophy, and/or muscle wasting), with liver ultrasonographic findings other than steatosis, or with increased liver transaminases >2–3 times the reference limits. In addition, 22 age- and sex-matched healthy volunteers who were reported to drink <15 g of EtOH per day, with normal liver function tests and standard hematological and biochemical tests, were analyzed. Before entering the study, each individual provided informed consent to participate, and the study was approved by the Ethics Committee of the University Hospital of Salamanca.

### 2.2. Experimental Design

Blood samples for hematological and biochemical tests, as well as both EDTA- and heparin-anticoagulated PB samples, were obtained from each subject between 9:00 and 10:00 AM under fasting conditions. EDTA-anticoagulated PB samples were used to separate plasma and isolate CD14+ monocytes to extract total ARN for PCR and miRNA expression analyses. Heparin-anticoagulated PB samples were used for the functional analyses described below: analysis of the M1/M2 phenotype after short-term culture of whole blood and after polarization of monocyte-derived macrophages (MDMs), and quantitation of soluble inflammatory cytokines after in vitro stimulation. RNA extraction, cDNA synthesis, qPCR, and cytokine analyses were performed on all samples after recruitment was completed, and the cases and controls were mixed in each respective plate.

### 2.3. CD14+ Monocyte Isolation and Polarization into Monocyte-Derived Macrophages

Peripheral blood mononuclear cells (PBMCs) from both EDTA- and heparin-anticoagulated PB specimens were isolated using FICOLL™ density gradient centrifugation within 1.5 h after blood draw. CD14+ monocytes were obtained from PBMCs using a magnetic bead-enrichment protocol (CD14 MicroBeads, Human, Miltenyi Biotech, Bergisch Gladbach, Germany) and platform (AutoMACS, Miltenyi Biotech) following the manufacturer’s instructions. CD14+ monocytes obtained from EDTA-anticoagulated PB samples were analyzed for purity, and total RNA was extracted using the miRNA easy Micro Kit (QIAGEN, Venlo, The Netherlands) and stored for further analyses. In turn, CD14+ monocytes from heparin-anticoagulated PB samples were seeded at a concentration of 1 × 10^6^/mL in RPMI medium enriched with 1% penicillin/streptomycin (10,000 U/10 mg per ml; Biochrom, Berlin, Germany), 2 mM L-Glutamine, and 10% fetal bovine serum for 6 days, of which the last 48 h were under M1, M2, or vehicle (M0) stimulation. M1 polarization was performed using 100 U/mL of human recombinant interferon (IFN)-γ (R&D Systems, Minneapolis, MN, USA) and 100 ng/mL of lipopolysaccharide (LPS) from *Escherichia coli* (055:B5 serotype, Sigma, Burlington, MA, USA), whereas M2 polarization was performed using 20 ng/mL of interleukin-(IL)-4 plus IL-13 (R&D Systems).

### 2.4. Analysis of M1/M2-Related Markers and TNFa-Producing Cells after Short-Term M1/M2 Stimulation

The production of the tumor necrosis factor (TNF)—a pro-inflammatory (M1-related) cytokine—and M2-related markers was analyzed on the monocyte surface membrane at the single-cell level, using a technique that combines the identification of monocytes and measurement of cytokine production in erythrocyte-lysed whole blood samples [4,18]. Briefly, three aliquots of heparinized PB samples were analyzed after short-term culture for 6 h at 37 °C in 5% CO_2_ and 95% humidity in a sterile environment in the presence of 10 μg/mL TACE inhibitor (Cytognos, Salamanca, Spain), which inhibits approximately 90% of all TNF-α secretion by PB monocytes and other cells [19], in order to allow its detection at the membrane level. Additionally, 100 ng/mL of LPS and 100 ng/mL of human recombinant IFN-γ were added as stimulatory agents to one aliquot (M1 stimulation), and 20 ng/mL of IL-4/IL-13 to another aliquot (M2 stimulation); a third non-stimulated aliquot under the other same culture conditions was also processed in parallel. After this short-term culture, all the three aliquots were stained with a combination of antibodies to unequivocally identify the classical monocytic population (CD45^++^, CD14^+/++^, CD16^−^, CD33^++^, and HLADR^+^), together with surface markers of M1 (TNF-α at the membrane level) and M2 (CD206 and CD209) polarization. As previously shown, the frequency of dead cells (monocytes or other cells) under the aforementioned culture conditions was irrelevant [18]; thus, the use of specific dyes to exclude dead cells was considered unnecessary. After staining, samples were measured on a FACSCanto II flow cytometer (Becton Dickinson, BD, San Jose, CA, USA) using the FACSDiva software (BD); data analysis was performed using the Infinicyt^®^ software (Cytognos).

The remaining (non-stained for flow cytometry) sample after the short-term culture, was used to isolate CD14+ monocytes for further RNA extraction. In turn, supernatants from erythrocyte-lysed whole blood samples after short-term M1/M2 stimulation were stored at −80 °C for the analysis of soluble cytokines.

### 2.5. Analysis of M1/M2 Phenotype in Monocyte-Derived Macrophages (MDMs) after 6-Day In Vitro Culture

After MDM in vitro polarization as previously described (M1 or M2), or left unstimulated with a negative control (M0), we analyzed at 6 d in vitro culture the cell surface expression on MDMs of M1/M2 markers (TNF-α, and CD206 and CD209, respectively) in a FACSCanto II flow cytometer, following the same staining procedure for flow cytometry as described above.

### 2.6. Quantitation of Soluble Levels of Inflamatory Cytokines

Basal plasma samples and supernatants from in vitro cultures (collected from whole blood samples after short-term cultures and at the end of stimulation of MDMs at 6 d after incubation) were used to measure the levels of soluble TNF-α (even in supernatants after inhibiting TACE, to measure remainder soluble levels), CXCL8/IL-8, IL-1β, IL-6, IL-10, and IL-12p70 in duplicate, using the Human Inflammatory Cytokine Cytometric Bead Array Kit (BD), according to the manufacturer’s instructions. Data were acquired using a FACSCanto II flow cytometer, and analysis was performed using the Flow Cytometric Analysis Program (FCAP) Array software v3.0 (BD).

### 2.7. RNA Isolation, cDNA Synthesis, and Real-Time PCR

Total RNA was extracted from isolated CD14+ monocytes and MDMs using the miRNA easy Micro Kit (QIAGEN), following the manufacturer’s instructions. Briefly, RNA was extracted using a phenol–chloroform mixture, precipitated in ethanol, and purified using RNase-free columns. RNA concentrations were determined using a spectrophotometer (NanoDrop, Thermo Fisher Scientific, Waltham, MA, USA) and a microfluidic chip (Agilent, Santa Clara, CA, USA). For mRNA expression analysis, complementary DNA (cDNA) was synthesized by reverse transcription using a commercial High-Capacity cDNA Reverse Transcription Kit (Applied Biosystems, Waltham, MA, USA) according to the manufacturer’s instructions. Relative quantitative real-time polymerase chain reaction (qPCR) was performed using SYBR Green PCR master mix (Applied Biosystems) and gene-specific primer sets, with actin as the reference gene. The PCR program for mRNA qPCR was 95 °C for 10 s, followed by 40 cycles of 95 °C for 15 s, and 60 °C for 30 s. For reverse transcription of total RNA-containing miRNAs, a primer-specific kit (miRCURY LNA Universal RT microRNA PCR, Exiqon, Vedbaek, Denmark) was used, and miRNA expression was determined by qPCR using miRNA-specific primers and the ExiLENT SYBR Green master mix, both from Exiqon, with U6 as the reference gene. qPCR experiments were performed in duplicate on a StepOnePlus™ Real-Time PCR System (Applied Biosystems), and primer specificity was verified by melt curve analysis. The threshold cycle (C_T_; number of cycles to reach the threshold of detection) was determined for each reaction, and gene expression was quantified using the 2^−ΔΔCt^ method [20].

### 2.8. miRNA Expression Analysis

miRNA expression was analyzed using specific arrays (Exiqon), following the manufacturer’s recommendations. Briefly, fluorescent reagents were reconstituted, and cDNAs were synthesized and labeled. Subsequently, the samples were heated at 95 °C in the dark, hybridized, and washed with the robotic HS 4800 Pro system (Tecan^®^, Männedorf, Switzerland). Fluorescence was scanned using a GenePix 4000 B microarray scanner (Molecular Devices, San Jose, CA, USA). Image processing was performed using Gene Prix Pro (v. 6.0), and data processing was performed using MEV (MultiExperiment Viewer) and EXpander 6.0. Functional enrichment analysis of deregulated genes, analysis of canonical pathways, correlation networks, and gene–gene and gene–miRNA interactions were defined using the Ingenuity Pathway Analysis (IPA) software (Ingenuity Systems; Qiagen China Co., Ltd., Shanghai, China) and the Database for Annotation, Visualization, and Integrated Discovery (DAVID) functional annotation.

### 2.9. Statistical Analysis

Mean values and their standard deviation (SD), median, and range, as well as the 25th and 75th percentiles, were calculated for each variable using the SPSS software program v26.0 (SPSS-IBM Statistics, Armonk, NY, USA). The statistical significance of the differences observed between controls and patients with heavy ethanol intake was determined using the Student’s *t*-test and the Mann–Whitney U-test for variables with a parametric and nonparametric distribution, respectively. GraphPad (GraphPad Software, San Diego, CA, USA) was used for graphical representation. As the data were not normally distributed, the Kruskal–Wallis test was applied for comparisons among >2 groups. When statistically significant differences were found (*p* < 0.05), the Dunn’s post hoc test was applied for comparisons among groups. The significance level was set at *p* < 0.05.

## 3. Results

### 3.1. Characteristics of the Study Cohort

The major characteristics of the patients and healthy controls included in this study are summarized in Table 1. No demographic differences in age or sex were found between the two groups. The mean daily alcohol consumption in the patient group was 207.7 g (SD = 208.7), and the mean duration of excessive alcohol intake was 260 months (SD = 181.4). Patients showed significantly increased serum levels of aspartate aminotransferase (AST), alkaline phosphatase (ALP), gamma-glutamyl transpeptidase (GGT), albumin, and ferritin. In addition, there was a significant increase in the mean corpuscular volume (MCV) of erythrocytes, as well as total leukocytes and neutrophils, and a decrease in the platelet count in the patients compared to controls.

### 3.2. Levels of Soluble Inflammatory Cytokines and the M1/M2 Phenotype and Production of Cytokines at the mRNA Level of CD14+ PB Monocytes after Short-Term Culture

**Figure 1:**
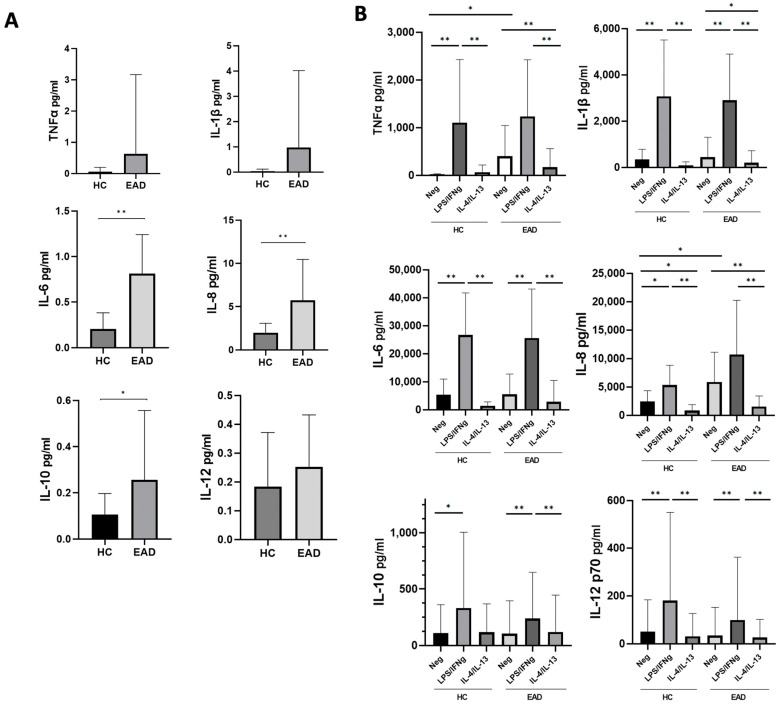
Soluble levels of inflammatory cytokines. (**A**) Basal (ex vivo) cytokine levels in plasma from healthy controls (HCs) and excessive alcohol drinkers (EADs). (**B**) Whole-blood cytokine levels from HCs and EADs upon short-term incubation without any stimulant/negative control (Neg), and with interferon (IFN)-γ plus lipopolysaccharide (LPS), or interleukin (IL)-4 plus IL-13. Data are presented as the mean and standard deviation. ** *p* < 0.01; * *p* < 0.05 by Mann–Whitney U-test (**A**), and Kruskal–Wallis test with Dunn’s post hoc test (**B**).

At basal (ex vivo) conditions, EADs showed increased plasma levels of all the cytokines analyzed in comparison to controls (Figure 1A), which were statistically significant for IL-6, IL-8, and IL-10 levels. After short-term whole-blood culture, unstimulated samples (negative control) from EADs showed significantly increased levels of TNF-α and IL-8 compared to those from healthy controls (Figure 1B). As expected, stimulation of whole blood with LPS/IFNγ induced a statistically significant increase in the production of the IL-1β, IL-6, and IL-12, as well as anti-inflammatory IL-10, compared to unstimulated whole blood samples in both HCs and EADs; the increase in soluble TNF-α and IL-8 was statistically significant only in controls. In turn, stimulation with IL-4/IL-13 induced a significant decrease in the production of both IL-1β and IL-8 in patients, and of IL-8 in controls, compared to unstimulated samples (Figure 1B).

**Figure 2:**
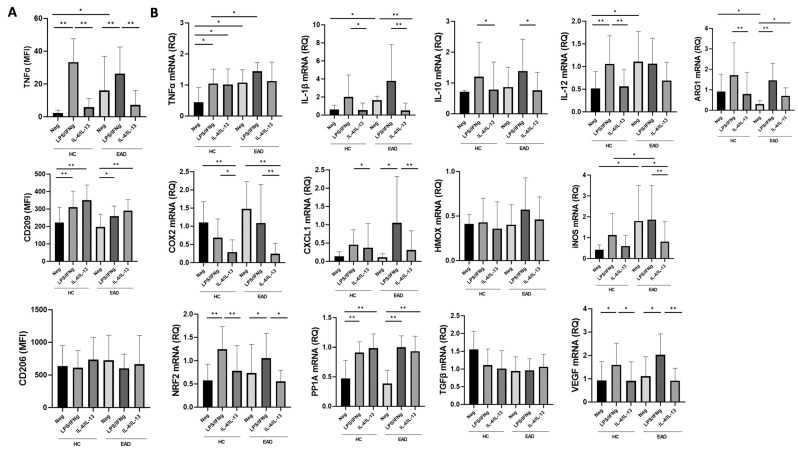
Expression of M1/M2 surface markers and relative expression of mRNA in blood CD14+ monocytes after short-term culture. (**A**) Surface expression of tumor necrosis factor (TNF)-a, CD209, and CD206 in peripheral blood CD14+ monocytes from healthy controls (HCs) and excessive alcohol drinkers (EADs) following short-term incubation without any stimulant/negative control (Neg), and with interferon (IFN)-γ plus lipopolysaccharide (LPS), or interleukin (IL)-4 plus IL-13. Results are expressed as mean fluorescence intensity (relative arbitrary units of fluorescence). (**B**) Relative mRNA expression in blood CD14+ monocytes from HCs and EADs following short-term stimulation with IFN-γ plus LPS or IL-4 plus IL-13. Results are presented as fold-changes relative to the control group. For both panels, data are shown as the mean (standard deviation). * *p* < 0.05, ** *p* < 0.01 by Kruskal–Wallis test with Dunn’s post hoc test. MFI: mean fluorescence intensity. RQ: Relative quantification.

In regards to the surface expression of M1/M2 markers after short-term whole-blood culture, PB CD14+ monocytes displayed significantly increased expression of TNF-α as a surface marker after blocking its secretion (Figure 2A) when comparing the unstimulated samples from EADs with those from controls. TNF-α expression was significantly increased in both groups after LPS/IFNγ stimulation, but without differences (*p* > 0.05) in the intensity of expression between patients and controls. No differences were found in CD206 expression between the groups, but CD209 levels were significantly increased after LPS/IFNγ, and particularly after IL-4/IL-13 stimulation, compared to unstimulated samples. As shown in Figure 2B, PB CD14+ monocytes from unstimulated samples of EADs had significantly increased mRNA expression of pro-inflammatory cytokines, such as TNF-α, IL-1β, IL-12, and inducible nitric oxide synthase (iNOS), and decreased mRNA expression of arginase, compared to unstimulated CD14+ monocytes from controls. LPS/IFNγ stimulation increased the mRNA expression of NF-E2-related factor-2 (Nrf2), protein phosphatase 1-alpha catalytic subunit (PP1A), and vascular endothelial growth factor (VEGF) in both cases and controls, as well as chemokine C-X-C-motif ligand 1 (CXCL1) in the patients. TNF-α and iNOS mRNA expression was significantly higher after stimulation with LPS/IFNγ in EADs than in the controls. Finally, IL-4/IL-13 stimulation significantly reduced iNOS and IL-1β mRNA expression in EADs compared to unstimulated samples, but this reduction was not observed in the controls.

### 3.3. M1/M2 Phenotype and Production of Inflammatory Cytokines at the mRNA Level by MDMs after Stimulation with LPS/IFNγ and IL-4/IL-13

**Figure 3:**
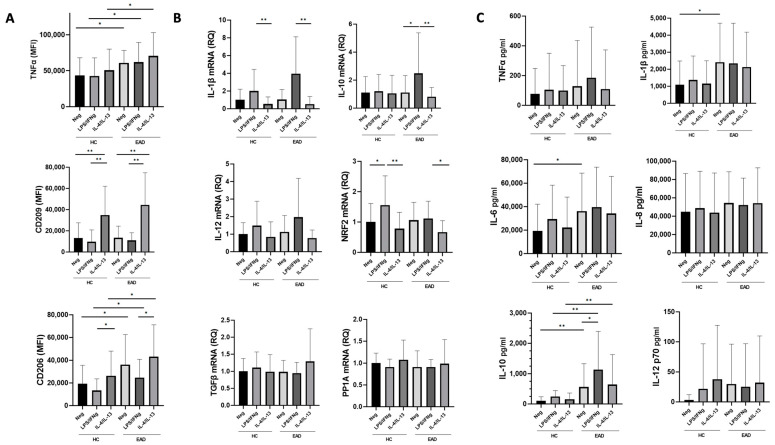
Expression of M1/M2 surface markers and relative expression of mRNA in monocyte-derived macrophages (MDMs). (**A**) Surface expression of tumor necrosis factor (TNF)-a, CD209, and CD206 in MDMs of healthy controls (HCs) and excessive alcohol drinkers (EADs) following stimulation without any stimulant/negative control (Neg), and with interferon (IFN)-γ plus lipopolysaccharide (LPS), or interleukin (IL)-4 plus IL-13. Results are expressed as mean fluorescence intensity (relative arbitrary units of fluorescence). (**B**) Relative mRNA expression MDMs of HCs and EADs following stimulation with IFN-γ plus LPS, or IL-4 plus IL-13. Results are presented as fold-changes relative to the control group. (**C**) Levels of soluble cytokines in supernatants from MDM cultures of HCs and EADs following stimulation with IFN-γ plus LPS or IL-4 plus IL-13. For all panels, data are shown as the mean (standard deviation). * *p* < 0.05, ** *p* < 0.01 by Kruskal–Wallis test with Dunn’s post hoc test. MFI: mean fluorescence intensity. RQ: relative quantification.

As shown in Figure 3, MDMs from EADs (both unstimulated and stimulated with either IFNγ plus LPS or IL-4/IL-13) displayed significantly higher levels of TNF-a and CD206 surface markers (Figure 3A) and produced increased supernatant levels of IL-10 (Figure 3C) compared to healthy controls. IL-6 and IL-1β supernatant levels were also higher in EADs than in controls, but only in unstimulated MDMs (Figure 3C).

MDMs from EADs stimulated with LPS/IFNγ showed increased IL-10 mRNA expression and supernatant IL-10 levels compared to unstimulated MDMs, but no significant differences between EADs and controls were found in TNF-a as a surface marker assessed by flow cytometry, or in the production of IL-1β, IL-6, IL-8, and IL-12 as assessed by supernatant levels or mRNA expression. CD209 expression was significantly increased in both EADs and controls after IL-4/IL-13 stimulation compared to that in unstimulated MDMs and those stimulated with LPS/INFγ. CD206 expression was significantly higher in both cases and controls when IL-4/IL-13 stimulation was compared to IFNγ plus LPS stimulation. MDMs from controls (but not from EADs) stimulated with IFNγ and LPS had higher NRF2 mRNA expression levels than unstimulated MDMs.

### 3.4. miRNA Analysis of CD14+ Monocytes

**Figure 4:**
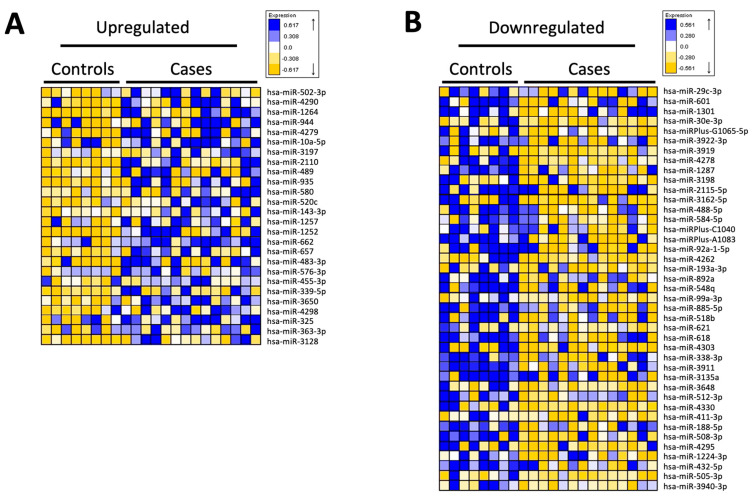
miRNA expression profiles of isolated CD14+ blood monocytes from excessive alcohol drinkers vs. healthy controls. Heatmap showing the expression of 67 differentially regulated human miRNAs in CD14+ monocytes isolated from peripheral blood. A total of 26 miRNAs were upregulated in the excessive alcohol drinking (case) group (**A**), and 41 miRNAs were downregulated (**B**).

A total of 67 human miRNAs were found to be differentially expressed between both groups (patients with excessive alcohol consumption and controls), with a threshold *p*-value of <0.05. Of these, 26 were upregulated in the patient group and 41 were downregulated compared with those in the control group (Figure 4A and Figure 4B, respectively). Analysis of the top genes with the greatest magnitude of differential expression associated with a *p*-value cut-off of 0.001 and fold-change ≥ |1.0| (up- or downregulated) showed a total of 18 top miRNAs (Table 2). Individual qPCR validation confirmed significant differences between cases and controls for all miRNAs except hsa-miR-10a-5p and hsa-miR-30a-3p, which showed no statistically significant differences between the groups. Regarding the relationship with oxidative stress, DAVID analysis used miRNAs differentially expressed between both groups to show that hsa-miR-483-3p, hsa-miR-520c-3p, and hsa-miR-30a-3p were associated with the NRF2-mediated oxidative stress response. The top molecular and cellular functions of the miRNAs identified by IPA analysis included hepatocellular carcinoma, liver hyperplasia/hyperproliferation, and liver cirrhosis (Table 3). All expression data were deposited in the Gene Expression Omnibus database (GEO; GSE239659).

## 4. Discussion

Our results, based on the analysis of cytokine levels in whole-blood samples and mRNA profile and surface marker expression on PB CD14+ monocytes, confirm that these latter cells from patients with excessive alcohol intake display a significantly increased spontaneous production of several pro-inflammatory cytokines compared with healthy individuals. This was further supported by the increased plasma levels (in EADs vs. HCs) of the IL-6 and IL-8 pro-inflammatory cytokines. The predominant pro-inflammatory profile here observed in EADs has been previously described in other reports including patients with heavy chronic ethanol intake [4,21], and it has been associated with alcohol-related organ damage. Regarding acute or binge ethanol consumption, some studies have also shown a pro-inflammatory effect [22,23], but other reports have suggested an anti-inflammatory effect of binge alcohol intoxication after a transient pro-inflammatory state [24]. Acute ethanol intake may thus have specific effects on immunity, different from those of chronic ethanol intake, and deserves further investigation.

In addition, our findings indicate that PB CD14+ monocytes from EADs have reduced expression of arginase mRNA (an M2-monocyte/macrophage marker), which reinforces the notion that a pro-inflammatory phenotype predominates in PB classical monocytes from these patients. As expected, stimulation with LPS/IFNγ significantly increased the production of pro-inflammatory cytokines, mRNA, and cell surface marker expression on monocytes from both EADs and controls, with significant differences being observed between both groups for TNF-α and iNOS mRNA expression (Figure 1B and Figure 2). TNF-α is a central cytokine in liver inflammation [4,21] and nitric oxide derived pro-oxidants have been associated with alcohol-induced hepatitis [25]. Although the lack of differences observed between cases and controls in other pro-inflammatory cytokines or in M1 markers expressed after stimulation with LPS/IFNγ may be due to the small sample size and the substantial inter-individual variability, these data fully confirm our previous findings. They show that, despite the increased production of inflammatory cytokines in the absence of any in vitro stimulant in individuals with chronic heavy ethanol intake compared to controls, no differences in cytokine levels were observed when PB CD14+ monocytes were stimulated with LPS/IFNγ [4]. Thus, these results confirm and extend on data from previous reports [4,18], suggesting the presence of in vivo activation and a blunted in vitro response to additional stimuli, which may be indicative of immune exhaustion.

We also observed that LPS/IFNγ stimulation induced production of the anti-inflammatory cytokine IL-10 as well as Nrf2 expression (Figure 1B and Figure 2B), which may modulate monocyte activation through antioxidant and anti-inflammatory pathways [11]. Indeed, Nrf2 is a master regulator of antioxidant responses and is able to interfere with the transcriptional upregulation of pro-inflammatory cytokines, such as IL-6 and IL-1β [10] as well as to induce macrophage polarization toward the M2 phenotype [26]. In addition, although M1 markers were predominantly expressed in circulating CD14+ monocytes, in vitro stimulation with LPS/IFNγ also induced CD209 expression (Figure 2A), and IL-10 levels were significantly increased in plasma along with pro-inflammatory cytokines (Figure 1). The finding of increased circulating levels of IL-10 among patients with chronic excessive alcohol intake has been previously described [16,27]. This cytokine, with anti-inflammatory and antioxidant properties, has protective roles in the development of liver disease and is produced in response to LPS stimulation [28,29]. Therefore, although predominantly M1, here we confirm the coexistence of both M1 and M2 phenotypes among CD14+ blood monocytes stimulated with LPS/IFNγ in our model of EADs without advanced liver disease. The presence of a hyperpolarized state with expression of both M1 and M2 markers has been already described in circulating peripheral blood mononuclear cells among alcoholic hepatitis patients as well as in animal models of ALD [13,30]. Therefore, the presence of M2 markers cannot be only related to the development of liver disease and the relevance and significance of this finding deserve further in-depth investigations to assess the impact of these phenotypes on ethanol-induced liver damage.

Analysis of MDMs also provided evidence of a predominantly pro-inflammatory profile, with increased surface marker TNF-a expression under all conditions in MDMs from individuals with excessive alcohol intake compared to controls, as well as elevated supernatant levels of certain pro-inflammatory cytokines (IL-6 and IL-1β) in unstimulated MDMs from EADs compared to controls (Figure 3A and Figure 3C, respectively). However, MDMs from individuals with excessive ethanol intake also exhibited heightened expression of the CD206 surface marker, as well as increased IL-10 supernatant cytokine levels. This concomitant presence of M2 markers along with a clear increase in M1 markers is consistent with previous reports on macrophages or Kupffer cells after alcohol intake or exposure [31,32]. Although comparisons between different experimental models are difficult, our results also support the hypothesis that ethanol impairs macrophage polarization to favor an M1 phenotype, which could be involved in alcohol-induced organ damage. This is also suggested by the lack of increase in Nrf2 mRNA expression in MDMs stimulated with LPS/IFNγ compared to that in unstimulated MDMs from cases, in contrast to the significant increase observed in controls (Figure 3B). Although the complex interaction between Nrf2 and NF-κB pathways during inflammation and oxidative stress in excessive ethanol intake and ALD requires further analysis, a role for Nrf2 in preventing M1 polarization has been described [33].

In addition, MDMs stimulated with LPS/IFNγ did not show a significant increase in pro-inflammatory cytokine production compared with unstimulated MDMs, although certain cytokines displayed a discernible trend without reaching statistical significance (Figure 3B,C). This observation may be attributed, at least in part, to macrophage exhaustion resulting from prolonged exposure to LPS associated with ethanol intake, as well as experimental conditions with prolonged ethanol exposure. Thus, our results suggest that both immune activation and immune exhaustion may occur in patients with excessive ethanol intake, which is potentially relevant for understanding the development of both organ damage and immunosuppression in these individuals.

In this regard, it is important to acknowledge that, although we used the common M1/M2 nomenclature, the M1/M2 model represents an in vitro paradigm that may only provide a useful guide for the in vivo setting, in which a continuum state of activation exists and macrophage functions are better represented as a network model [34]. Although our protocol to induce M1/M2 polarization in vitro has been used in previous studies to assess monocyte/macrophage phenotype, our results do not fully reflect the complex nature of macrophage activation in vivo, where a multitude of stimuli are concurrently present and interacting [8,35]. In any case, the different markers employed in this study may be helpful for reliable identification of pro-inflammatory and anti-inflammatory phenotypes [8,9]. In turn, the use of three different methods for the assessment of the M1/M2 functional monocytic/macrophage profiles (surface M1/M2 marker expression levels and cytokines measured as soluble mediators in plasma specimens and culture supernatants, as well as at the mRNA expression level) has clear advantages compared to the limited data from immune system function that provides the single measurement of cytokine levels, without a link to the specific cell types that produce the secreted cytokines [36,37]. Despite using several methods, differences in individual peaks of cytokine production and lack of complete correlation between the methods, along with individual variability and the limited sample size, make it difficult to reach definite conclusions in this regard. For instance, a recent study found that patients with alcohol use disorders had a significant increase in IL-6 concentrations but a reduction in TNF-α approximately 3 h after acute alcohol ingestion [38]. In our study, all EADs with chronic ethanol intake showed active alcohol consumption in the last 48 h, and we found an immune profile consistent with increased levels of inflammatory cytokines, as previously reported [4,21]. Another limitation of our manuscript is the absence of an analysis of LPS levels, which could have been useful for correlating with M1/M2 markers and for making comparisons between groups.

Regarding miRNA analysis of isolated CD14+ circulating monocytes, it is difficult to compare our data with those of other studies, owing to methodological differences and the fact that similar studies in the field of alcohol use disorders in humans have been performed in other tissues or plasma [39]. Although our analysis is preliminary, this is the first study in which mRNA analysis was performed in human circulating monocytes isolated from blood.

However, several differences in miRNA expression found in our study, have also been previously associated with a pro-inflammatory phenotype, such as upregulation of the pro-inflammatory miR-92a-3p [40] or downregulation of the anti-inflammatory miR-885-5p, which has been described to promote an activation of the NF-κB pathway in other settings [41]. Indeed, upregulation of miR-339-5p or miR-489-3p may be associated with an anti-inflammatory phenotype, since these miRNAs have been associated with the inhibition of alcohol-induced brain inflammation or TLR4/NF-κB signaling in psoriasis, respectively [42,43]. These miRNAs have been associated with other cell signaling pathways, potentially linking inflammation with oxidative stress, such as hsa-miR-339-5p [44], or carcinogenesis, such as hsa-miR-92a-3p [45]. In any case, the differential expression of miRNAs potentially associated with both M1 and M2 phenotypes is consistent with our results after cytokine and mRNA analysis, and has also been described after miRNA analysis of Kupffer cells isolated from rats fed a chronic ethanol diet, which was confirmed in PBMCs from patients with alcoholic hepatitis [30]. Of note, while the latter study showed decreased expression of miR-143-3p in Kupffer cells, here we found upregulation of this miRNA in isolated CD14+ blood monocytes, highlighting the presence of conflicting results in this type of analysis, which could also be explained by differences between murine Kupffer cells and circulating human monocytes. Taken together, our results provide preliminary results on the miRNA profile of circulating monocytes in the blood of EADs, which should be validated in additional studies.

In summary, our data reinforce previous studies that indicate a predominantly pro-inflammatory profile of circulating monocytes in chronic excessive drinkers. Furthermore, our findings extend previous knowledge by demonstrating a mixed M1/M2 phenotype in both circulating monocytes and monocyte-derived macrophages, along with a specific miRNA profile. Despite the limitations of our study as outlined above, our results reveal an interplay of pro- and anti-inflammatory effects, which may hold potential for modulating immune system activation and ameliorating ethanol-induced organ damage in scenarios of chronic excessive alcohol consumption

## 5. Conclusions

Our data strongly indicate that circulating CD14+ blood monocytes and MDMs from individuals with chronic excessive alcohol intake exhibit a predominantly pro-inflammatory phenotype with concomitant expression of some M2 anti-inflammatory and antioxidant markers associated with a blunted in vitro response to LPS/IFNγ, which is suggestive of immune exhaustion.

## Figures and Tables

**Table 1:** Characteristics of excessive alcohol drinkers (EADs) and healthy controls.

Variable	EAD Patients (n = 20)	Controls (n = 22)	*p*-Value
Age (years) Sex (M/F) *	46.6 (10.5) 15/5 (75%/25%)	42.3 (14.1) 15/7 (68.2%/31.8%)	0.27 0.63
Total Bilirubin (mg/dL)	0.7 (0.8)	0.6 (0.3)	0.48
AST (U/L)	43.0 (38.0)	20.4 (8.5)	0.02
ALT (U/L)	49.3 (56.2)	23.3 (8.2)	0.05
ALP (U/L)	86.0 (35.7)	52.3 (12.8)	0.001
LDH (U/L)	171.4 (42.9)	185.6 (64.4)	0.42
GGT (U/L)	194.3 (303.8)	23.5 (20.7)	0.02
Proteins (g/dL)	7.3 (0.6)	7.5 (0.4)	0.24
Albumin (g/dL)	6.0 (1.4)	4.7 (0.2)	0.001
Ferritin (ng/mL)	498.9 (562.4)	151.3 (112.7)	0.01
Hemoglobin (g/dL)	15.2 (1.5)	15.3 (1.3)	0.74
Hematocrit (%)	42.0 (9.9)	43.5 (7.4)	0.57
Erythrocyte MCV (fL)	95.5 (6.8)	89.2 (4.0)	0.001
Erythrocyte MCH (pg)	33.1 (2.8)	30.5 (1.6)	0.001
Leukocytes (×10^3^ cells/μL)	7.6 (1.9)	6.3 (1.4)	0.001
Neutrophils (×10^3^ cells/μL)	4.4 (1.7)	3.3 (1.0)	0.008
Lymphocytes (×10^3^ cells/μL)	2.2 (0.6)	2.3 (0.6)	0.47
Platelets (×10^3^ cells/μL)	214.9 (73.9)	262.5 (67.8)	0.03
Total cholesterol (mg/dL)	212.9 (41.0)	220.8 (35.7)	0.51
Triglycerides (mg/dL)	126.4 (68.6)	115.4 (50.4)	0.55
Prothrombin activity (%)	90.8 (9.3)	94.9 (6.1)	0.11
APTT (seconds)	37.3 (5.1)	34.7 (2.2)	0.07
Fibrinogen (mg/dL)	372.1 (111.8)	311.0 (57.8)	0.05
D-Dimer (μg/mL)	0.5 (0.3)	0.4 (0.7)	0.70

Results are expressed as mean (standard deviation) or as * number of cases (percentage). AST, aspartate aminotransferase. ALT: alanine aminotransferase. ALP: alkaline phosphatase. EAD: excessive alcohol drinker. F: female. LDH: lactate dehydrogenase. GGT: gamma-glutamyl transferase. M: male. MCV: mean corpuscular volume. MCH: mean corpuscular hemoglobin. APTT: activated partial thromboplastin time.

**Table 2:** Top miRNAs with the greatest magnitude of differential expression in patient vs. control blood cells (*p*-value cut-off of < 0.001 and a fold-change of  ≥|1.0|).

miRNAs	Fold-Change
hsa-miR-483-3p	+2.906
hsa-miR-95	+1.834
hsa-miR-339-5p	+1.498
hsa-miR-143-3p	+1.293
hsa-miR-489	+1.219
hsa-miR-501-3p	+1.198
hsa-miR-92a-3p	+1.128
hsa-miR-10a-5p	+1.093
hsa-miR-885-5p	−2.364
hsa-miR-3176	−1.721
hsa-miR-188-5p	−1.610
hsa-miR-512-3p	−1.560
hsa-miR-515	−1.427
hsa-miR-455-3p	−1.422
hsa-miR-30a-3p	−1.374
hsa-miR-1224-3p	−1.370
hsa-miR-421-3p	−1.361
hsa-miR-584-5p	−1.333

Positive fold-change values indicate upregulated miRNAs, and negative values indicate downregulated miRNAs.

**Table 3:** Top molecular and cellular functions of miRNAs identified by Ingenuity Pathway Analysis (IPA).

Category	Functions Annotation	*p*-Value	Molecules	# Molecules
Hepatocellular carcinoma	Liver cancer	3.45 × 10^−5^	hsa-miR-143-3p hsa-miR-192-5p hsa-miR-193a-3p hsa-miR-29b-3p hsa-miR-483-3p hsa-miR-515 hsa-miR-92a-3p	7
Liver hyperplasia /Hyperproliferation	Liver cancer	3.45 × 10^−5^	hsa-miR-143-3p hsa-miR-192-5p hsa-miR-193a-3p hsa-miR-29b-3p hsa-miR-483-3p hsa-mir-515 hsa-miR-92a-3p	7
Liver hepatitis	Chronic hepatitis B	5.83 × 10^−2^	hsa-miR-143-3p	1
Renal inflammation	lupus nephritis	1.47 × 10^−1^	hsa-miR-92a-3p	1
Renal nephritis	lupus nephritis	1.47 × 10^−1^	hsa-miR-92a-3p	1
Liver cirrhosis	Cirrhosis of liver	2.22 × 10^−1^	hsa-miR-143-3p	1

## Data Availability

The data presented in this study are available upon request from the corresponding author. The data are not publicly available for ethical reasons.
